# Editorial: Advances in biotechnology-based breeding of medicinal plants

**DOI:** 10.3389/fpls.2023.1228951

**Published:** 2023-06-21

**Authors:** Mohsen Niazian, Davoud Torkamaneh, Mohsen Hesami

**Affiliations:** ^1^ Département de Phytologie, Université Laval, Québec City, QC, Canada; ^2^ Institut de Biologie Intégrative et des Systèmes (IBIS), Université Laval, Québec City, QC, Canada; ^3^ Centre de recherche et d’innovation sur les végétaux (CRIV), Université Laval, Québec City, QC, Canada; ^4^ Institute Intelligence and Data (IID), Université Laval, Québec City, QC, Canada; ^5^ Department of Plant Agriculture, University of Guelph, Guelph, ON, Canada

**Keywords:** medicinal plants, biotechnology, plant breeding, *in vitro* micropropagation, gene transformation

As green chemical factories, medicinal plants contain a wide range of bioactive compounds crucial for biomaterial industries. Despite their high economic value, medicinal plants are in the last ring of domestication syndrome and have been neglected for many years by plant breeders. In recent years, plant scientists have tried to compensate for these shortages by developing different strategies, especially faster biotechnology-based methods (BBMs) to conserve and improve these valuable but neglected plants. For the first step, the information and awareness of endangered medicinal plants is very important. Kakkar et al. reviewed all available information related to the nomenclature and classification, endangerment, plant morphology, ploidy, secondary metabolites, drug pharmacokinetics, conservation, and omics-based computational studies in *Aconitum* genus. The presented information is very valuable in terms of conservation of endangered economically important poisonous mountainous medicinal plant species in this genus.

The assessment of the genetic background of valuable medicinal plants is the second step to improve medicinal plants and schedule efficient breeding programs. In this context, the research article by Wei et al. applied the complete chloroplast genome sequencing and phylogenetic study to distinguish three Gaoben-related medicinal plants, including *Ligusticum sinense*, *L. jeholense*, and *Conioselinum vaginatum*, which are similar in morphology and are difficult to distinguish from each other by the commonly used DNA barcodes. The authors proved that their method is very valuable in the identification of the mentioned Gaoben-related medicinal materials as they found highly variable region (ycf2-trnL and accD-ycf4) within the chloroplast genomes of *C. vaginatum*, *L. sinense*, and *L. jeholense*.

Increasing valuable bioactive compounds is the third step in the improvement of medicinal plants. The use of elicitors is one of the common methods to increase valuable secondary metabolites. This can be done *ex vitro* or *in vitro*. *Ex vitro* application of elicitors is the simplest and most cost-effective method. Mubeen et al. present a valuable protocol to increase valuable secondary metabolites of *Silybum marianum* using Aspergillus niger, methyl jasmonate (MeJA) and silver nanoparticles (AgNPs) elicitors in hydroponic medium. Based on their results, MeJA was considered as the best elicitor, which leads to the highest level of total phenolics and SOD activity in *S. marianum*. Classical statistical methods have low accuracy to optimize the *in vitro* application of elicitors in medicinal plants. The application of machine learning algorithms is one of the powerful and effective methods for optimizing the tissue culture conditions of medicinal plants. Different machine learning algorithms have been applied to modeling various *in vitro* culture types of medicinal plants. García-Pérez et al. present a machine learning-based model to decipher the critical factors involved in the response to elicitation in cell suspension cultures of medicinal *Bryophyllum*. The authors revealed that the genotype-dependent role of salicylic acid was more than methyl jasmonate elicitor and the established model was efficient to predict the production of flavones, isoflavones, flavanones, stilbenes, and flavanols the in eliciting cell suspension cultures.

The combination of innovative isolation techniques with biotechnology-based breeding methods provides an excellent platform to increase valuable bioactive components of medicinal plants. Finally, we appreciate the contribution from Jang et al. who conducted a series of experiments to establish an optimized method to isolate ginseng exosomes with high purity. In this research article, authors applied ultracentrifugation and ExoQuick methods and reported that the combination of ultracentrifugation and ExoQuick led to the improved purity and the colloidal stability of isolated ginseng exosomes. Their established protocol would be applicable to isolate high-purity and high-stability exosomes of other valuable medicinal plants.

## Author contributions

All authors listed have made a substantial, direct and intellectual contribution to the work, and approved it for publication.

